# Genome-wide identification of Mg^2+^ transporters and functional characteristics of DlMGT1 in *Dimocarpus longan*

**DOI:** 10.3389/fpls.2023.1110005

**Published:** 2023-02-02

**Authors:** Xinmin Lv, Shilian Huang, Jing Wang, Dongmei Han, Jianguang Li, Dongliang Guo, Haifeng Zhu

**Affiliations:** ^1^ Key Laboratory of South Subtropical Fruit Biology and Genetic Resource Utilization, Ministry of Agriculture, Key Laboratory of Tropical and Subtropical Fruit Tree Research of Guangdong Province, Guangzhou, China; ^2^ Key Laboratory of Crop Harvesting Equipment Technology of Zhejiang Province, Jinhua Polytechnic, Jinhua, China

**Keywords:** magnesium transporter, longan, transcription factors, genomics, cis-acting elements

## Abstract

Longan (*Dimocarpus Longan*) is one of the most important fruit crops in Southern China. Lack of available Mg in acidic soil conditions is a limitation to further increasing longan yield. Magnesium transporter (MGT/MRS2) mediates the uptake, transport, and redistribution of Mg2+ in higher plants. To understand the role of MGTs family members in longan Mg deficiency. We identified and analyzed the protein characteristics, phylogeny, expression changes, subcellular localization, and transcriptional regulation of DlMGTs members. The results showed that, twelve DlMGTs are localized in the cell membrane, chloroplast, and nucleus. The evolutionary differences in MGTs between herbaceous and woody species in different plants. The DlMGTs promoters contained many cis-acting elements and transcription factor binding sites related to the hormone, environmental, and stress response. Subcellular localization assays showed that DlMGT1 localizes in the cell membrane of *Arabidopsis* protoplasts. The candidate transcription factor DlGATA16, which may regulate the expression of DlMGT1, was localized in the nucleus of tobacco leaves. Dual luciferase analysis demonstrated that DlGATA16 is a potential factor regulating the transcriptional activity of DlMGT1. In this study, we identified and analyzed DlMGTs on a genome-wide scale and the subcellular localization and interaction of DlMGT1 and DlGATA16, which has important implications for further functional analysis studies of MGTs and the use of MGT for longan genetic improvement.

## Introduction

Magnesium (Mg) is the fourth most essential plant nutrient and the most abundant divalent cation in cells (Hermans et al., 2013). Mg plays a critical role in the growth and development of higher plants, including the synthesis of chlorophyll (Wilkinson et al., 1990), assimilation of carbon dioxide in photosynthesis (Sreedhara and Cowan, 2002), distribution of carbohydrates, and metabolism of energy (Clarkson and Hanson, 1980). Available magnesium deficiency will lead to the blocking of chlorophyll synthesis in plants, which in turn causes leaf abscission. Long-term Mg deficiency leads to a decrease in dry weight, which can seriously affect the growth and development of plants (Hermans et al., 2013). Because Mg has a relatively small ionic radius and a large hydration radius, it is weakly bound to the soil and plant root surface and is easily leached from the soil (Maguire and Cowan, 2002). For a long time, due to high temperatures and rainfall, the Mg-containing minerals in red loam soils in Southern China have been subjected to weathering and leaching, resulting in lower available Mg content in the soil (Sánchez and Logan, 1992; Nawaz et al., 2010). Therefore, Mg deficiency is a significant cause of low crop productivity and poor product quality in acidic soil areas.

Mg^2+^ uptake and metabolism depend on MGTs (Smith et al., 1993; Schock et al., 2000; Graschopf et al., 2001). Family members of MGT have been identified in *Arabidopsis* (Gebert et al., 2009), rice (Saito et al., 2013), sugarcane (Wang et al., 2019), maize (Li et al., 2016), and rapeseed (Zhang et al., 2019). The functions of several MGTs have been intensively studied in *Arabidopsis* and rice (Chen et al., 2009; Bose et al., 2011; Conn et al., 2011; Chen et al., 2012; Ishijima et al., 2012). To date, 10 AtMGTs have been reported, all of which can restore the Mg^2+^ uptake capacity of the yeast-deficient strain *cm66*; therefore, all AtMGTs possess the ability to transport Mg^2+^ (Schock et al., 2000; Li et al., 2001). However, only OsMRS2-1/3/6/9 transports Mg^2+^ in rice (Saito et al., 2013). Different MGTs have different affinities to Mg^2+^. While AtMGT1/2/10 have high affinity (Schock et al., 2000; Li et al., 2001; Conn et al., 2011) and AtMGT3/7/9 have low affinity (Whiteman et al., 2008; Chen et al., 2009; Gebert et al., 2009), AtMGT5 has dual-affinity to Mg^2+^ (Li et al., 2008). Members of AtMGTs also have distinct subcellular localisations and biological functions. AtMGT1 is localised in the cell membrance and involved in the uptake of Mg^2+^ in roots, and its transport activity correlates with aluminium tolerance (Li et al., 2001; Deng et al., 2006). The functions of mitochondrial membrane-localised AtMGT5 (Li et al., 2008), as well as endoplasmic reticulum-localised AtMGT9 (Chen et al., 2009) and AtMGT4 (Li et al., 2001; Li et al., 2008), are related to pollen development, and these AtMGTs are expressed at different stages of pollen development. Vesicular membrane-localised AtMGT2 and AtMGT3 are involved in intracellular Mg^2+^ partitioning in leaf sarcomeres (Schock et al., 2000; Alexandersson et al., 2004; Carter et al., 2004; Whiteman et al., 2008; Conn et al., 2011) AtMGT6 is localised on the cytoplasmic membrane. Its expression in roots maintains plant growth under low Mg^2+^ conditions and that in aboveground parts mainly maintains Mg^2+^ homeostasis in leaves under high Mg^2+^ stress (Mao et al., 2014). AtMGT10 is localised in the chloroplast membrane and is involved in the transport and metabolism of Mg^2+^ in the chloroplast (Drummond et al., 2006).

Longan is a critical fruit tree of tropical and subtropical regions in China, primarily planted in Guangdong and Fujian provinces. Its fruit is nutritionally and medicinally valuable (Lai et al., 2000; Li and Chen, 2021; Wang et al., 2022). Through detailed measurement and evaluation of soil nutrients in these areas, it was found that Mg deficiency under acidic soil conditions and application in agricultural production are the nutrient-limiting factors inhibiting further improvement of longan yield (Dong and Zhang, 2010; Zhang et al., 1999). Mg^2+^ uptake and metabolism depend on MGTs (Smith et al., 1993; Schock et al., 2000; Graschopf et al., 2001). However, the identification and functional studies of MGT family members in longan have not been reported. In this study, we performed genome-wide identification of DlMGTs based on the completion of high-quality genome sequencing of longan (Lin et al., 2017; Wang et al., 2022). Furthermore, we characterised longan gene structure, chromosome distribution, gene duplication, cis-acting elements, transcription factor binding sites, and phylogenetic relationships. In addition, we analysed the subcellular localisation of DlMGT1 and DlGATA16 and the interaction between DlGATA16 and DlMGT1 promoters. Our results will benefit the functional studies of MGTs in longan, which is vital for the genetic improvement of longan.

## Materials and methods

### Plant materials and Mg deficiency treatments

*Arabidopsis* culture. *Arabidopsis* seeds were sterilised using 75% ethanol and 8% NaClO. The sterilised seeds were vernalised at 4°C for three days. The vernalised seeds were sown onto the surface of a moist, sterile substrate and placed in an artificial climate chamber. The environmental factor controller of the artificial climate incubator was adjusted to short daylight (10–13 h light at 23°C/11–14 h dark at 20°C), low light (50–75 μEm^-2^S^-1^) conditions, and relative humidity was maintained at 40–65%. Four weeks later, *Arabidopsis* leaves were used for protoplast preparation.

Cultivation of tobacco. Tobacco seeds were sterilised in EP tubes containing 75% alcohol and 0.1% L of mercury. Sterile tobacco seeds were sown on MS medium and cultured in the dark at 25°C for four days. The light culture was used for 30 d for subcellular localisation and luciferase assay.

Burying of fresh longan seeds in wet sand to promote germination. Germinated longan was cultured in complete Hoagland nutrient solution for two weeks to adapt to the culture conditions. The culture conditions were as follows: 14 h light at 26°C/10 h dark at 26°C. Longan seedlings that could grow normally in Hoagland nutrient solution were treated for Mg deficiency. The control treatment consisted of longan seedlings cultured in complete Hoagland nutrient solution, and the Mg deficiency treatment consisted of longan seedlings cultured in Hoagland nutrient solution lacking Mg. The protocol was repeated three times for each treatment.

### Identification and bioinformatic analysis of the DlMGTs gene family

Longan genomic and proteomic data were obtained in the SapBase (http://www.sapindaceae.com/) database. The 10 *Arabidopsis* MRS2/MGT proteins obtained from the TAIR database (https://www.arabidopsis.org/) were used as query sequences for homology matching in the longan proteome data using the BLAST function of TBtools (Chen et al., 2020) software. ExPASy (https://web.expasy.org/protparam/), TMHMM 2.0 (https://services.healthtech.dtu.dk/service.php?TMHMM-2.0), signalP 3.0 (https://services.healthtech.dtu.dk/service.php?signalP-3.0), and CELLO (http://cello.life.nctu.edutw/) were used to analyse protein physicochemical properties, transmembrane structural domains, signalling peptides, and subcellular localisation of DlMGTs members. The gene structure of DlMGT members was visualised using Gene Structure Display Server 2.0 (http://gsds.gao-lab.org/). Conserved motifs of DlMGTs were identified using MEME (https://meme-suite.org/meme/doc/meme.html), with minimum and maximum motif lengths set to 6 and 200, respectively, and e ≤ 1e^-5^. The WebLogo 3 website (https://weblogo.threeplusone.com/) was used to generate sequence flags for the conserved domains of DlMGTs. Multiple sequence comparisons of MGTs from different plant species were created using the MUSCLE Align algorithm in the MEGA 7 software. Phylogenetic trees were constructed using the maximum likelihood method under the parameters of 1,000 bootstrap replicates. The phylogenetic trees were visualised and embellished using Evolview (http://www.evolgenius.info/evolview/). The promoter sequences (approximately 2,000 bp upstream of the start codon) of all genes in longan were downloaded from the SapBase database, and the promoter sequences of all DIMGT members were extracted using TBtools. Finally, promoter sequences were submitted to the PlantCARE (http://bioinformatics.psb.ugent.be/webtools/plantcare/html/) and PlantTFDB (http://plantregmap.gao-lab.org/binding_site_prediction.php) databases for cis-acting progenitor and trans-acting factor binding site prediction.

### Chromosomal localisation and gene duplication analysis of DlMGTs

Covariance analysis was performed and visualised using MCScanX (Wang et al., 2012) and Circos software (Krzywinski et al., 2009), respectively. All longon (cv. Shixia) sequences were compared using the local BlastP program with an e-value of 1e^-10^, num-thread value of 8, and num-alignments value of 5. The BlastP results were compared using the genome annotation file (Dimocarpus_longan_SX.genome.gff3) as input, and MSCanX was used to generate covariance and tandem files and to screen DlMGTs for nodes and tandem repeat genes, respectively.

### Reverse transcription quantitative PCR

Total RNA was extracted using the Aidlab RN53 EASYspin Plus RNA kit. First strand cDNA was synthesised using HiScript ^®^ II Q RT SuperMix for qPCR (Vazyme, R232-01). Next, qRT-PCR was performed using an ABI QuantStudioTM 6 Flex system (ThermoFisher Scientific) with a SensiFASTTM SYBR Lo-ROX Kit (Bioline, BIO-94005). The thermocycler protocol was as follows: pre-denaturation, 1 min at 95 °C, 40 cycles at 95 °C for 15 s, 56°C for 15 s, and 72 °C for 45 s. Primers used are listed in **Supplemental Table S1**.

### Subcellular localisation of DlMGT1 and DlGATA16

Total RNA was extracted from the leaves of longan (cv. Shixia) using the TIANGEN RNAprep Pure Plant Plus kit, and first-strand cDNA was synthesised using the PC64-THERMOscript RT kit. The CV21-Zero Background pTOPO-TA/Blunt Cloning kit and KOD FX were used to clone the full-length CDSs of DlMGT1 and DlGATA16. The primer sequences are detailed in **Supplemental Table 1**. The expression vectors were constructed using Thermo Fisher GeneArt Gibson Assembly. The pAN580-GFP and pRI101-eGFP expression vectors were linearised with restriction endonuclease BamHI-HF (NEB, R136V) and purified for recovery, and the vector construction procedure was performed according to the kit manufacturer’s instructions. A 5 µL reaction system was used, consisting of 2.5 µL Mix, 1.5 µL linearised vector, and 1 µL target fragment, and the ligated products were immediately transformed using *Trans5α* Chemically Competent Cell by thermal excitation method at 50°C for 30 min. The cells were incubated overnight at 37°C. Single colonies were isolated and cultured in LB liquid medium containing 50 µg/mL Amp, and positive clones were screened by PCR and sequenced.

Fifteen *Arabidopsis* leaves of good growth condition were selected at the rosette stage and cut into 0.5 mm width shreds with a razor. The leaves were put into 10 mL enzymatic digestion solution (1.5% Cellulase R10 and 0.4% Macerozyme R10) for 2 h. The enzymatic digestion products were filtered through a 200-mesh cell sieve, transferred to a 50 mL centrifuge tube, and centrifuged at 100 g for 2 min, and the resulting precipitate consisted of *Arabidopsis* protoplasts. An appropriate amount of rinsing solution (MMg solution) was taken to re-solubilise the protoplasts. Using the PEG-Ca^2+^-mediated method (Shen et al., 2014), the control vector pAN580-GFP and the constructed fusion expression vector pAN580-DlMGT1-GFP were transformed into *Arabidopsis* leaf protoplasts. The results were observed after 12 h of dark incubation in a laser confocal microscope (FLUOVIEW FV3000, OLYMPUS).

The pRI101-DlGATA16-eGFP plasmid was transferred into *Agrobacterium tumefaciens* GV3101, coated with kanamycin-resistant plates, and the monoclonal clone was isolated from a liquid medium containing 50 µg/mL Kan LB at 28°C and incubated at 200 rpm for 12 h. The culture was centrifuged at 4,000 g for 5 min to collect the bacterium and resuspended with 10 mmol·L^-1^ MgCl_2_. The mixture containing 120 *Agrobacterium* was injected by applying pressure to the lower epidermis of tobacco leaves while using a 1 mL syringe without a needle. After two days of incubating the injected tobacco plants in low light, tobacco leaves that were injected were extracted, and slides were made to observe the leaves under a laser confocal microscope (FLUOVIEW FV3000, OLYMPUS); the above operation was also repeated using an empty vector-transformed *Agrobacterium* as the control.

### Luciferase analysis of DlGATA16 and DlMGT1 promoters

In this experiment, the dual-luciferase reporter assay system was used to analyse the regulatory role of the transcription factor DlGATA16 on the DlMGT1 promoter. The pUC18-DlGATA16-3HA and pGreenII0800-PDlMGT1-LUC plasmids were cloned and constructed using the RT-qPCR method outlined in Section 4. The primers are detailed in **Supplemental Table 1**. Each combination of pUC18-3HA, pGreenII0800-LUC, pUC18-DlGATA16-3HA, and pGreenII0800-PDlMGT1-LUC was transferred into *Agrobacterium* strain GV3101, and tobacco leaves were injected as outlined in Section 5. The injected tobacco was incubated in an artificial climate chamber in low light for a second. The reverse side of the leaf was coated with 1 mmol·L^-1^ of firefly fluorescein (Promega) and placed in the dark for 5 min. The leaves were then cut and subjected to *in vivo* fluorescence detection in a NightShade LB 985 *In Vivo* Plant Imaging System.

## Results

### Identification of DlMGTs family members

We used BLAST to match sequences, E values ≤10^-7^, homologous to AtMGTs in the genome of the longan Shixia cultivar (cv.) (Li et al., 2022) using TBtools software (Chen et al., 2020). We finally obtained 12 DlMGTs after removing two members (Dil.06g013540.1 and Dil.11g004700.1) lacking a transmembrane domain (**Table 1**). The DlMGTs were named based on their phylogenetic distances from AtMGTs (**Supplemental Figure 1**). The coding sequence length of DlMGTs ranged from 240 to 1,509 bp, and the amino acid (aa) sequence length ranged from 80 to 503 aa. The theoretical molecular weights of DlMGTs range from 9178.43 to 55827.44 Da, with isoelectric points between 4.90 and 9.10. Approximately 75% of DlMGTs were unstable hydrophilic proteins. None of the DlMGTs proteins contained signal peptide sequences. DlMGT9.1, DlMGT3, DlMGT10, DlMGT1, and DlMGT7 contained two transmembrane domains, and DlMGT4.2 contained one transmembrane domain. DlMGT9.1, DlMGT10, DlMGT1, DlMGT7, and DlMGT4.2 localised in the inner cell membrane, DlMGT3 in the cytoplasmic membrane, DlMGT9.4 and DlMGT9.2 in the chloroplast, and the remaining DlMGTs localised in the nucleus (**Table 1**).

**Table 1:** Basic information on the DlMGT gene family members.

Gene name	Gene ID	CDS/bp	GC	Chromosomelocation	Size/aa	Molecular weight/Da	Theoretical pI	Instability index	Transmembrane domain	Signal peptide	Subcellular localization
DIMGT9.1	Dil.01g030430.1	1113	510	Chrl:39816771..39822105 (-)	371	41475.6	5.79	42.65	2	NO	endomembrane system
DIMGT3	Dil. 10g013390.1	1245	500	Chr10:22007711.22013332 (+)	415	46072.75	5.26	38.1	2	NO	plasma membrane
DIMGT4.1	Dil.11g003500.1	834	415	Chr11:6878859.6880097 (-)	278	30528.73	5.15	45.68	0	NO	nucleus
DIMGT10	Di1.13g008140.1	1344	613	Chr13:6519399.6528031 (-)	448	49780.16	5.4	61.06	2	NO	endomembrane system
DIMGT9.6	Di1.07g004250.1	240	99	Chr7:9889369..9890436(-)	80	9178.43	5.46	45.13	0	NO	nucleus
DIMGT9.4	Di1.15g017640.1	369	159	Chrl5:15398393..154016990 (-)	123	13741.02	6.27	44.89	0	NO	chloroplast
DIMGT9.2	Di1.05g011330.1	453	212	Chr5:20808698.208101430 (+)	151	17021.47	9.1	58.77	0	NO	chloroplast
DIMGT9.5	Di1.06g013610.1	369	178	Chr6:17476283..17477706(+)	123	13847.72	6.29	45.55	0	NO	nucleus
DIMGT1	Dil.03g025940.1	1470	642	Chr3:38601383..38614318(-)	490	55519.97	5.24	51.59	2	NO	endomembrane system
DIMGT7	Di1.04g006370.1	1173	480	Chr4:4754222..4758700( (-)	391	44319.45	4.9	35.48	2	NO	endomembrane system
DIMGT4.2	Dil. 11g016850.1	1509	715	Chrl1:23598308.23603050 (+)	503	55827.44	4.84	45.1	1	NO	endomembrane system
DIMGT9.3	Dil.08g018320.1	303	140	Chr8:2912698229128391 (+)	101	11323.81	8.71	30.04	0	NO	nucleus

### Phylogenetic and gene replication analysis of DlMGTs

**Figure 1:**
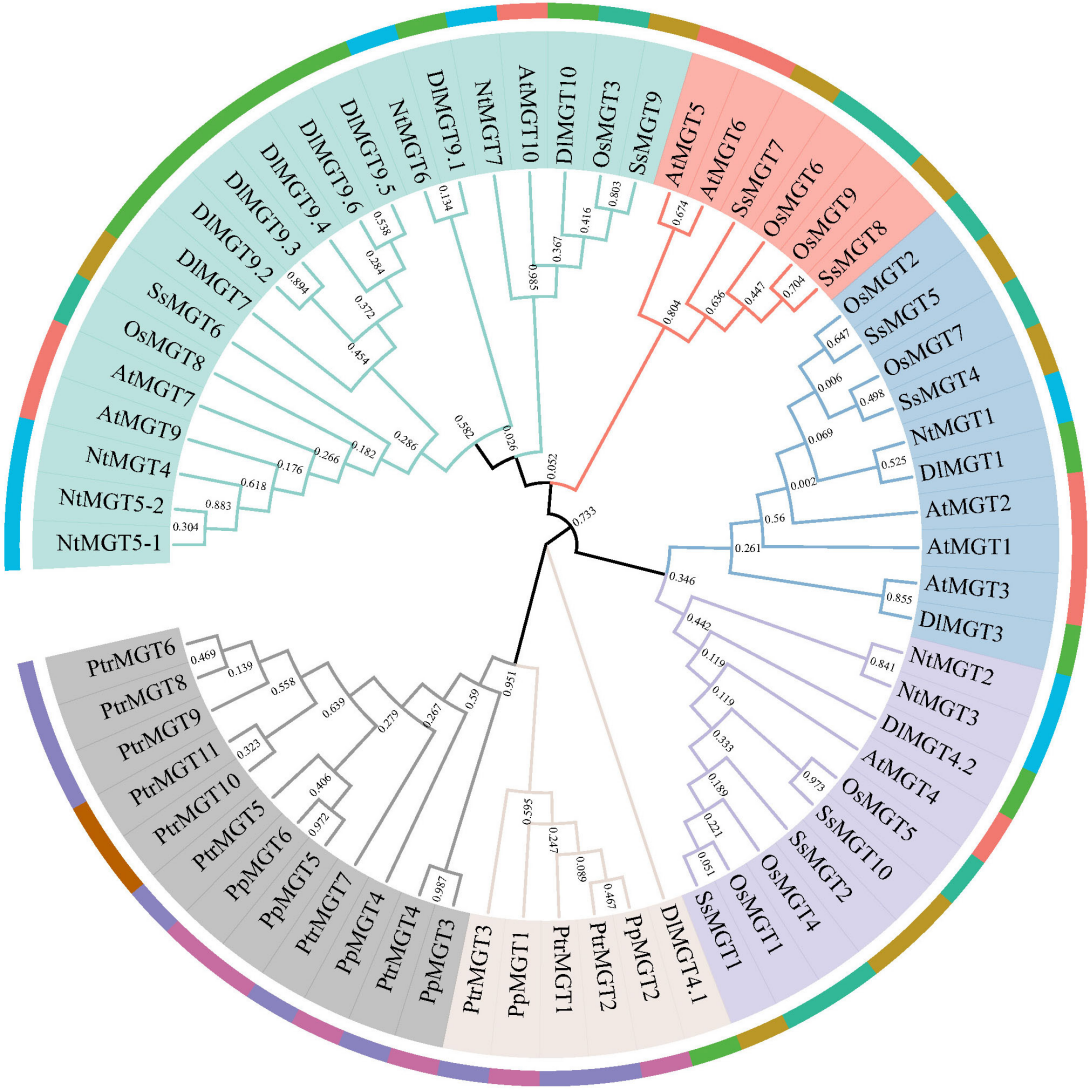
Phylogenetic tree for longan, *Arabidopsis*, tobacco, rice, poplar, peach, and sugarcane MGT proteins. The phylogenetic tree was created using MEGAX using the neighbour-joining approach after MGT proteins from the longan (*Dimocarpus longan* Lour., DlMGTs), *Arabidopsis* (*Arabidopsis thaliana*, AtMGTs), tobacco (*Nicotiana rustica* L., NtMGTs), poplar (*Populus trichocarpa* Torr. and Gray, PtrMGTs), sugarcane (*Saccharum spontaneum*, SsMGTs), peach (*Prunus persica*, PpMGTs), and rice (*Oryza sativa*, OsMGTs) were initially aligned using ClustalW. A total of 1,000 bootstrap replications were used.

To elucidate the phylogenetic relationships of MGTs, we constructed maximum likelihood trees from *Arabidopsis*, tobacco, sugarcane, poplar, peach, rice, and longan. As shown in **Figure 1**, the MGTs were divided into six branches, two of which had no DlMGTs members. PtrMGTs and PpMGTs were grouped into the same branch, suggesting that the evolution of MGTs in herbaceous plants may be more complex and have occurred earlier than in woody plants. In the phylogenetic tree, the MGT evolutionary relationship between longan and herbaceous plants was closer, suggesting that the evolution of longan MGT genes may have occurred earlier than that of poplar and peach MGT genes.

**Figure 2:**
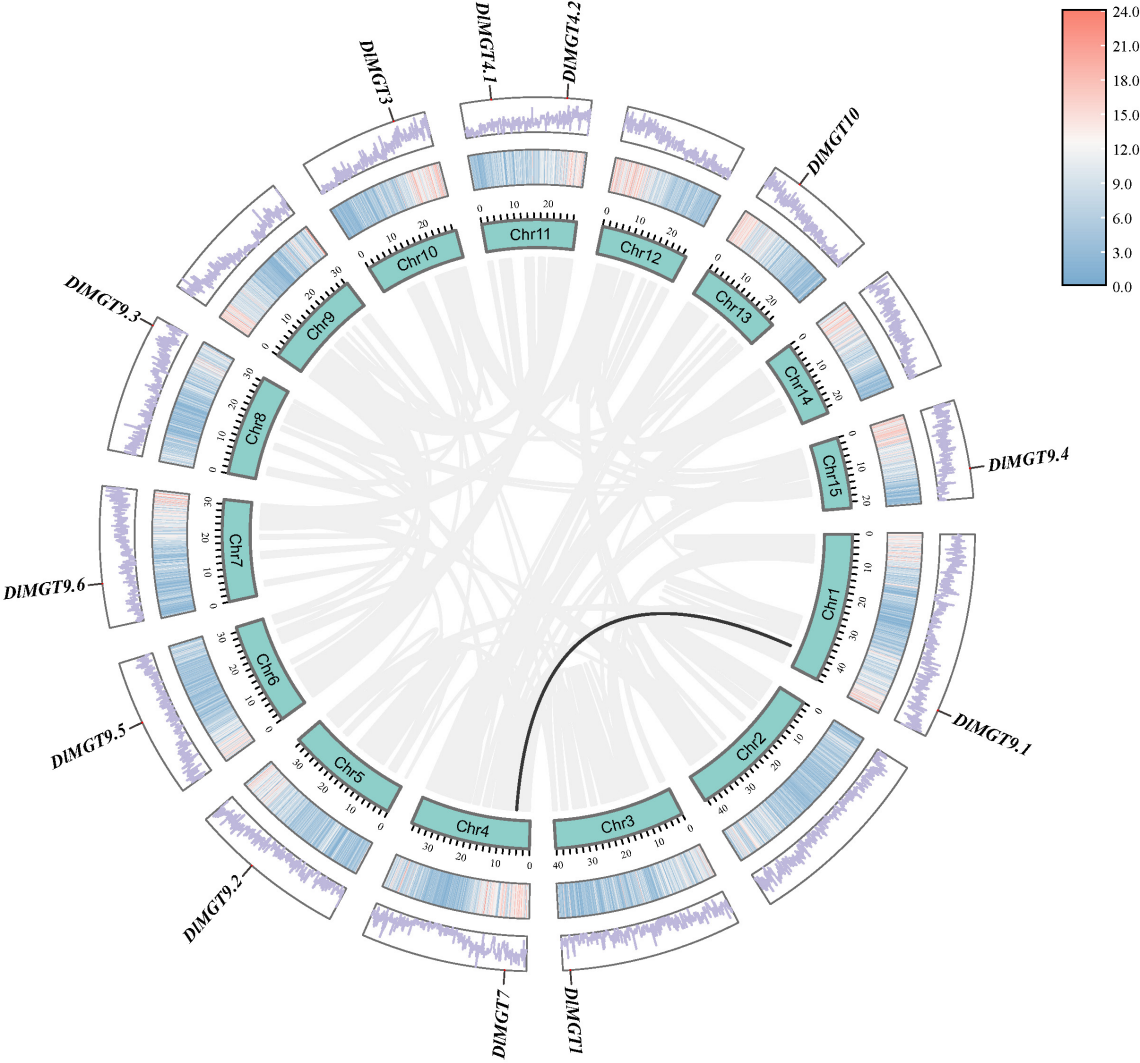
Chromosomal location and collinearity analysis of *DlMGT* family genes. Cyan boxes represent chromosomes. Segmental duplication genes are connected with black lines.

We mapped the gene locations to understand the DlMGT distribution on longan chromosomes (**Figure 2**). DlMGTs are distributed on the longan chromosomes randomly and uniformly. Each chromosome harboured one DlMGT member, and chromosome 11 harboured two DlMGT members. In our collinearity relationship analysis, only one pair of duplicated genes, DlMGT9.1 and DlMGT7, was obtained (**Figure 2**), indicating that DlMGTs have few duplication events in the genome.

### Analysis of DlMGTs gene structure and conserved motifs

**Figure 3:**
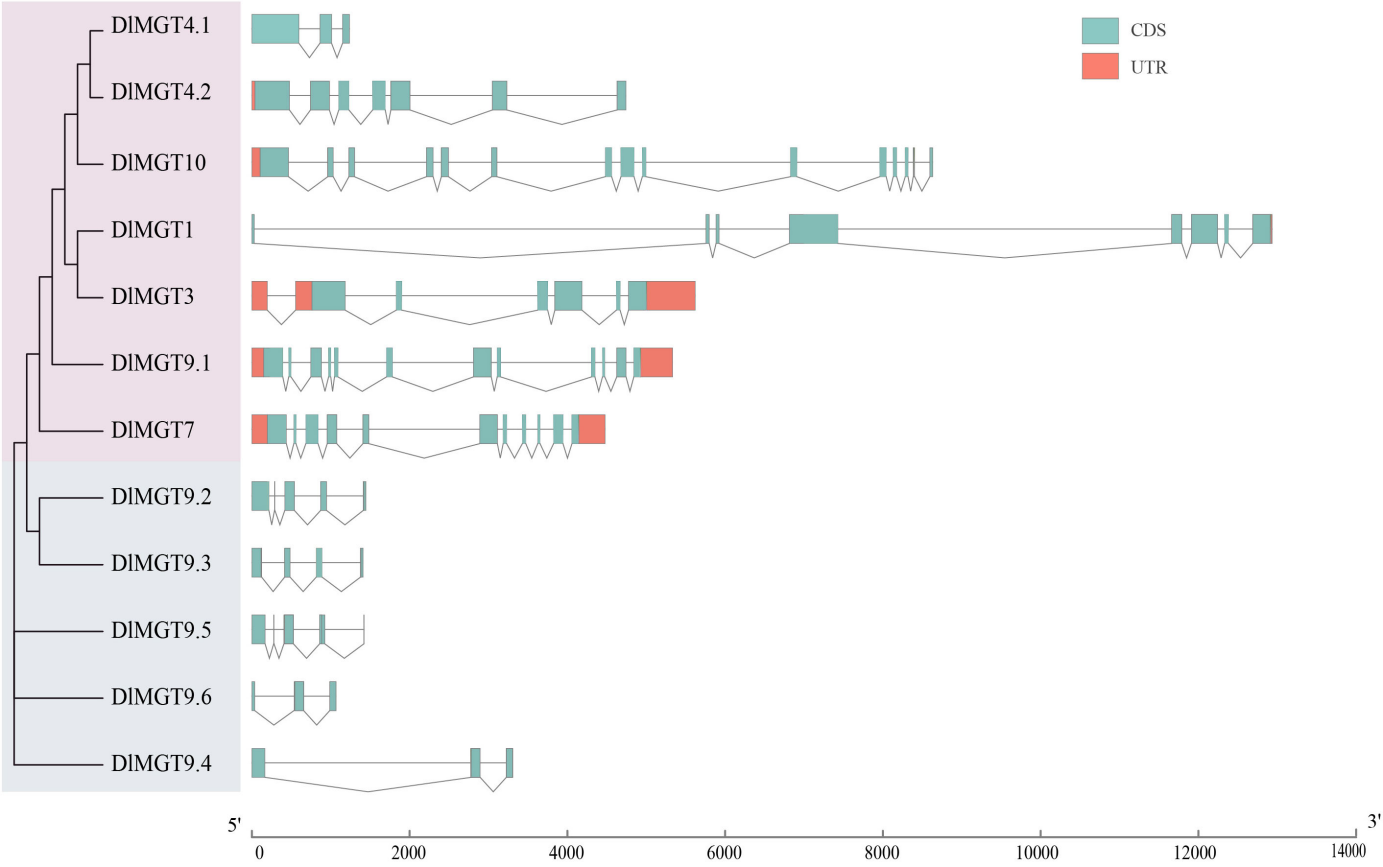
Gene structure diagram of *DlMGTs*. UTRs are indicated by orange boxes and introns by black lines; cyan boxes represent CDS. The sizes of exons and introns can be estimated using the scale at the bottom.

We analysed the phylogenetic relationships among different members of DlMGTs and characterised the distribution of exons and introns for each DlMGT member. Gene structure analysis showed that all DlMGTs contained introns, with DlMGT10 exhibiting the highest number of introns (14 introns), followed by DlMGT9.1 and DlMGT7, which contained 11 and 10 introns, respectively. The second subfamily members are similar in gene structure; their gDNA lengths are less than 2,000 bp and they all lack UTR regions (**Figure 3**).

**Figure 4:**
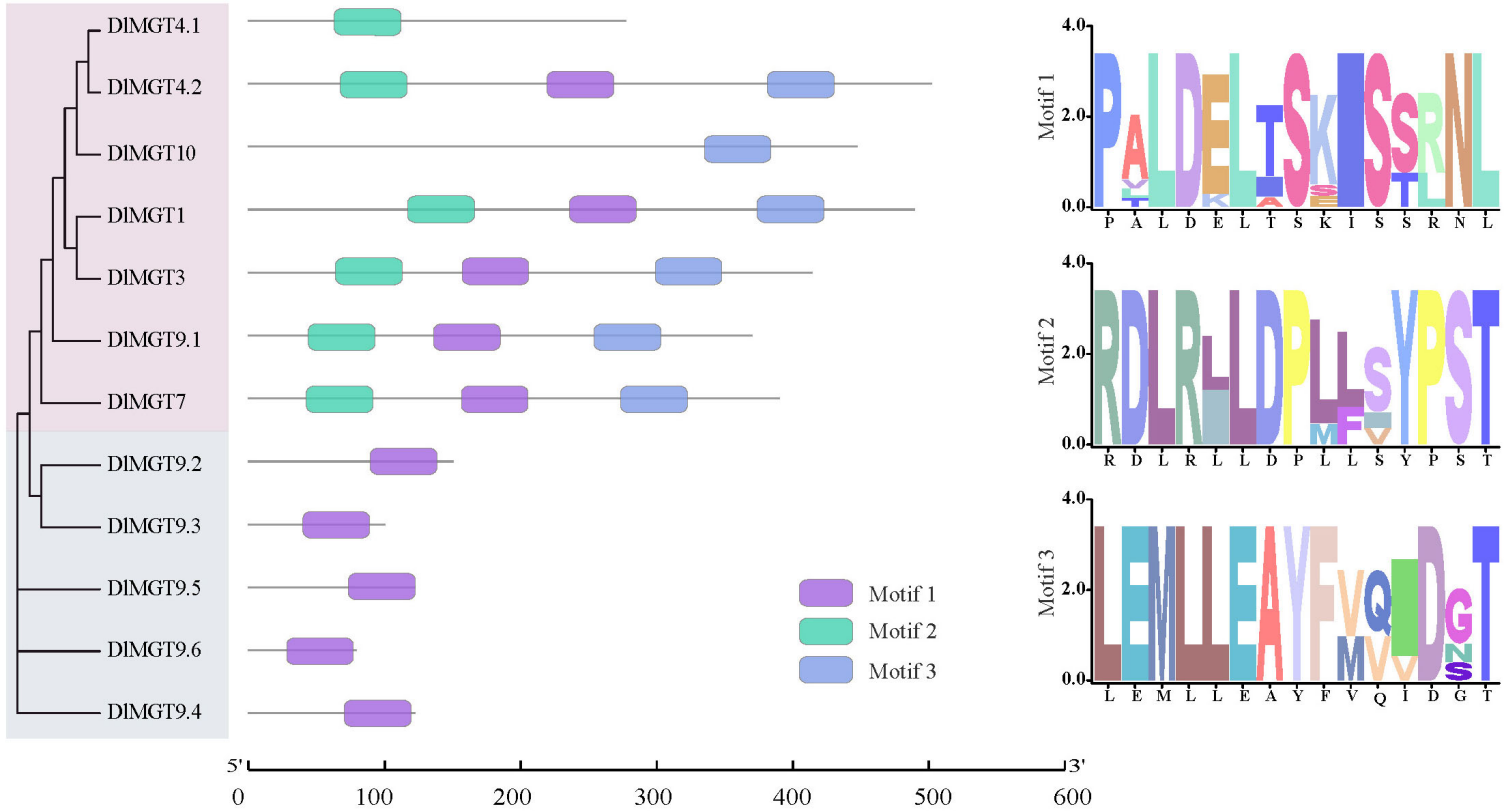
Conserved motifs of DlMGT proteins. The purple, cyan, and light blue boxes represent the conserved motifs 1, 2, and 3, respectively. The right side indicates the conserved histidine motifs of DlMGT proteins.

Using the MEME website, we identified three conserved motifs in DlMGT members and drew a LOGO diagram for each motif. All DlMGTs subfamily members contained motif 2. Furthermore, all members of the first subfamily contained motifs 1, 2, and 3, except for DlMGT4.1, which only had motif 2. In contrast, all members of the second subfamily contained only motif 1. Motifs 2 and 3 are more conserved in the DlMGTs family than motif 1 (**Figure 4**).

### Expression analysis of DlMGTs

**Figure 5:**
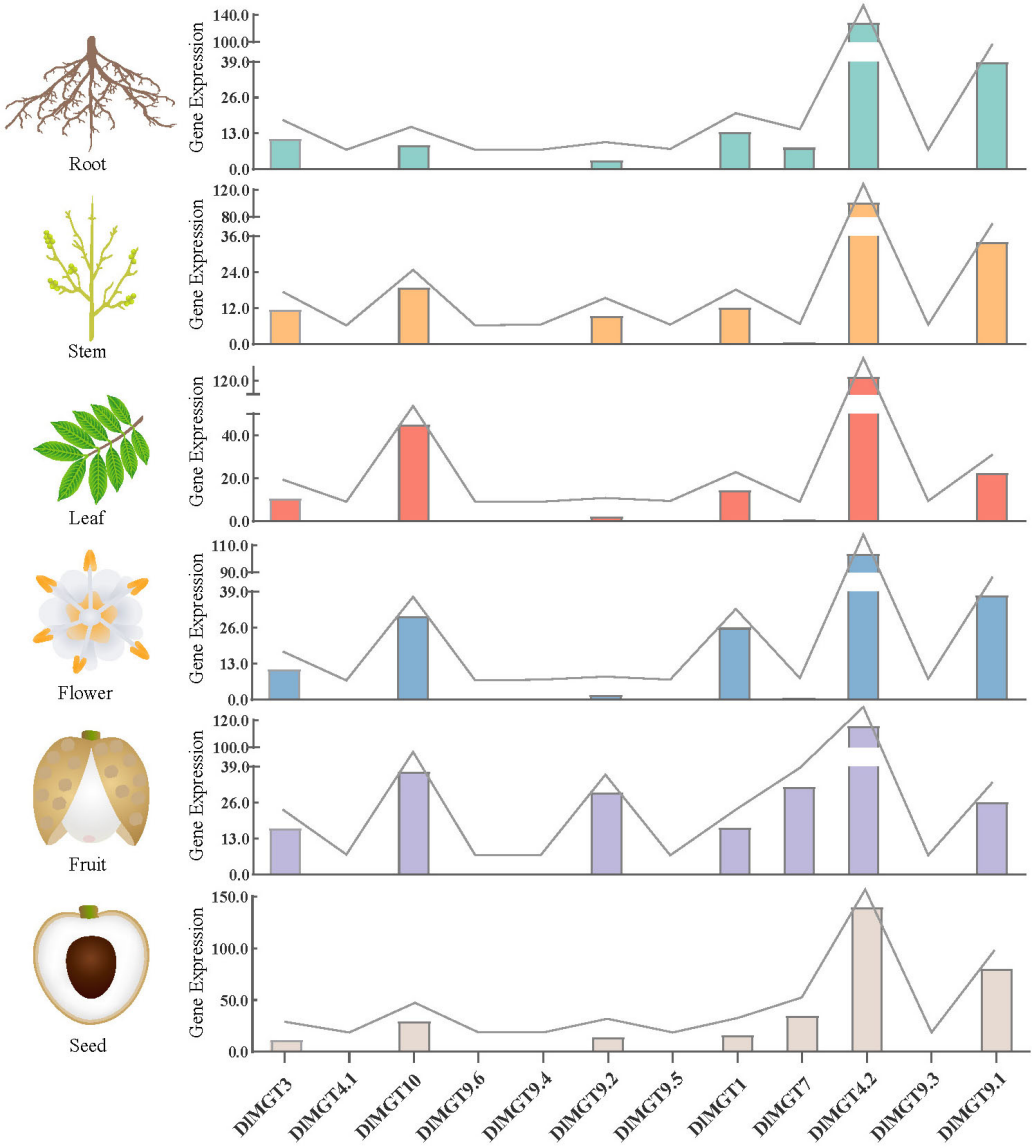
Differential expression of *DlMGTs* in different tissues of longan based on FPKM values.

We analysed the expression levels of *DlGMTs* in longan roots, stems, leaves, flowers, fruit, and seeds using longan transcriptomic data (BioProject NO. PRJNA329283) from the NCBI website. The results showed that *DlMGT4.1*, *DlMGT9.6*, *DlMGT9.4*, *DlMGT9.5*, and *DlMGT9.3* were not expressed in any longan organs (**Figure 5**). The expression levels of *DlMGTs* also differed between organs. *DlMGT9.2* was highly expressed in fruit but hardly expressed in flowers and leaves. *DlMGT7* was expressed in roots, fruit, and seeds but not leaves and flowers. Moreover, *DlMGT3*, *DlMGT10*, *DlMGT1*, *DlMGT4.2*, and *DlMGT9.1* were expressed in all organs. *DlMGT4.2* exhibited the highest expression across all organs, followed by *DlMGT10* and *DlMGT9.1*.

**Figure 6:**
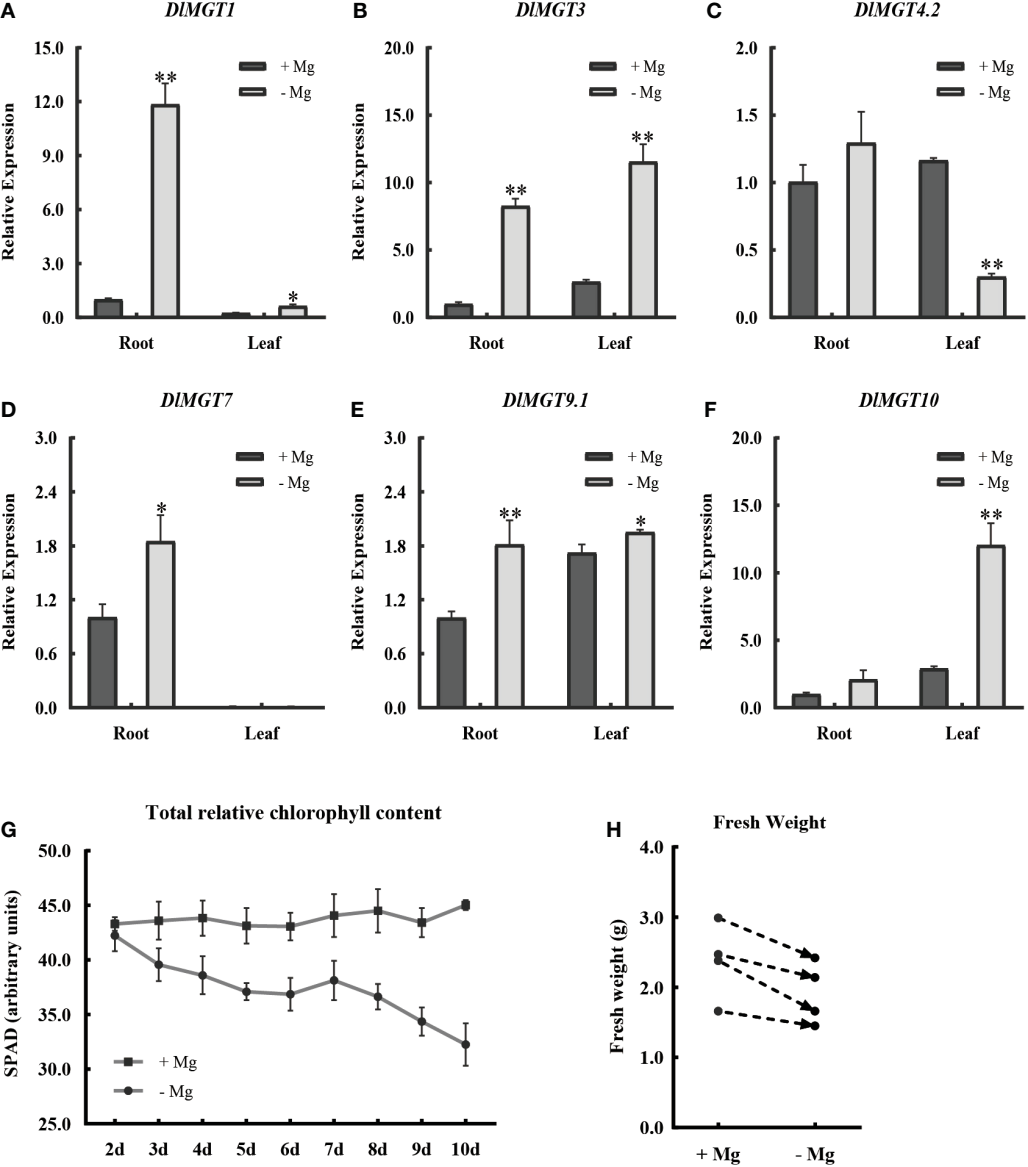
Changes in *DlMGTs* expression, chlorophyll content, and fresh weight of longan under Mg deficiency treatments. **(A-F)**. Changes in the expression of DlMGTs in roots and leaves of longan seedlings under Mg sufficiency and deficiency treatment conditions after 48 h. **(G)**. Changes of total relative chlorophyll content in longan leaves under continuous Mg-deficient treatment. **(H)**. Changes of fresh weight of longan seedlings after 10 d of Mg sufficiency and deficiency treatments. *difference is significant. **difference was extremely significant.

In order to explore the effect of Mg deficient stress on longan, we examined the expression of plasma membrane-localised *DlMGTs* in the roots and leaves of longan seedlings after 48 h under Mg- sufficient and deficient treatments. We also examined the chlorophyll content and fresh weight under Mg deficiency stress. The results showed that all *DlMGTs* responded to Mg deficiency stress (**Figures 6A-F**). Under Mg deficiency stress, different *DlMGTs* had different expression levels in roots and leaves. For example, *DlMGT1* and *DlMGT7* were mainly expressed in roots, and *DlMGT10* was mainly expressed in leaves. Compared with that under the Mg-sufficient treatment, the total chlorophyll content of longan decreased with extension of the Mg-deficient treatment (**Figure 6G**). After Mg deficiency treatment for 10 d, the fresh weight of four repeated longan seedlings decreased (**Figure 6H**). This showed that Mg deficiency affected chlorophyll synthesis, resulting in weakened photosynthesis, and ultimately limited the growth of longan.

### Analysis of cis-acting elements in DlMGTs promoter

**Figure 7:**
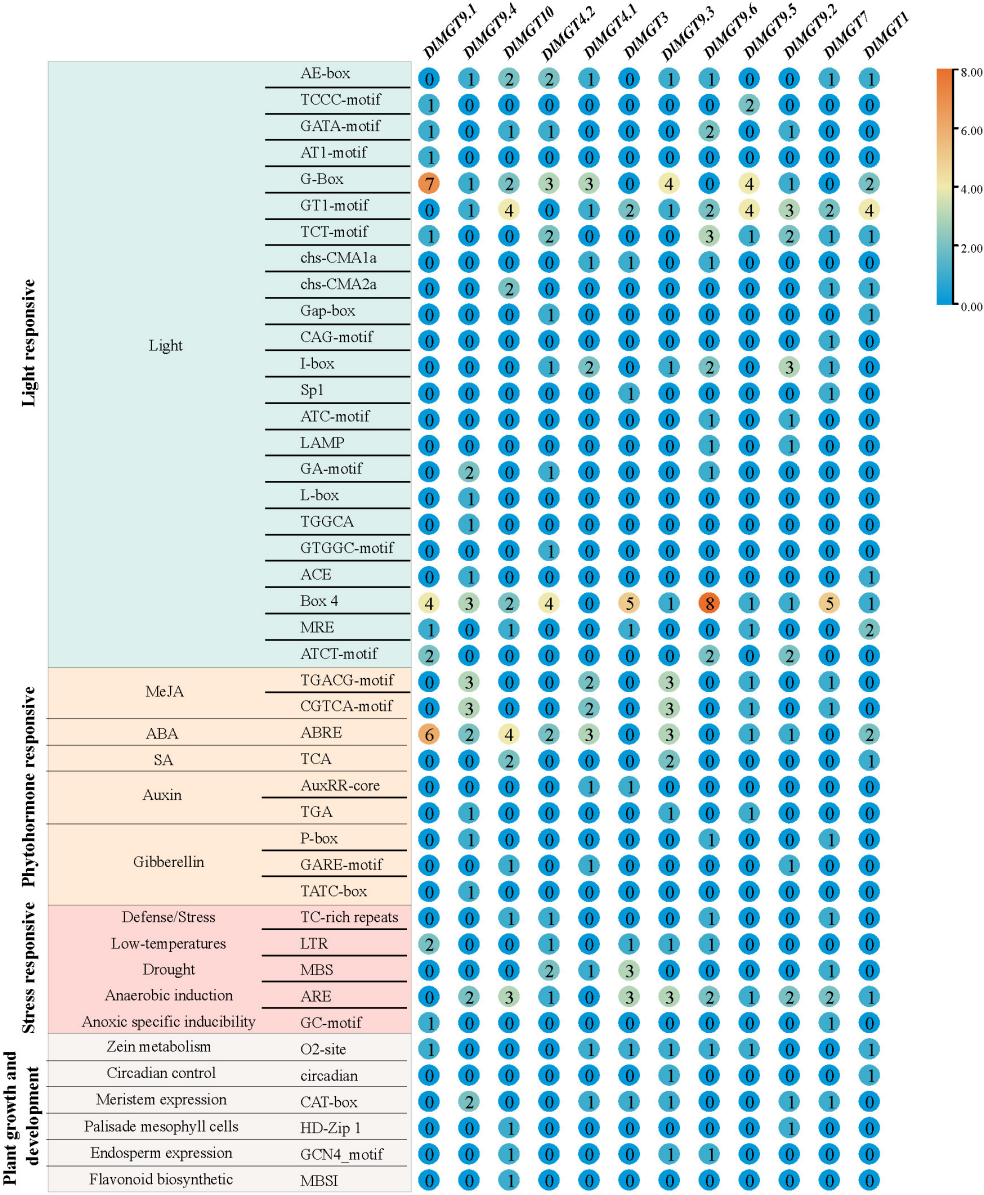
Distribution of cis-acting elements in promoters of *DlMGT* gene family members.

We predicted the cis-regulatory elements of *DlMGT* promoters using PlantCARE to identify the types of transcriptional regulation in *DlMGTs*. The cis-acting elements of *DlMGT* promoters were divided into four categories: light, hormone, stress response, and elements related to plant growth and development (**Figure 7**). Light-responsive elements exist in all *DlMGT* promoters. In addition, among the hormone response elements, abscisic acid exhibited the highest number of response elements (24), followed by methyl jasmonate (MeJA) with 20 response elements. Cis-elements (TGACG and CGTCA motifs) involved in the MeJA signalling response were found in the promoters of five *DlMGTs*. All *DlMGT* promoter regions contained at least one hormone response element (**Figure 7**).

The promoters of *DlMGTs* also contained many stress response elements. The most significant number of these were anaerobically induced elements. The promoters of *DlMGT9.1* and *DlMGT4.1* did not contain the anaerobic induction-related element, which was found in all other *DlMGTs*. In addition, we identified several cis-acting elements related to plant growth and development, including circadian control (circadian), meristem expression (CAT-box), maize alcohol soluble protein metabolism (O2site), palisade mesophyll cells (HD-Zip 1), endosperm expression (GCN4_Motif), and flavonoid biosynthetic (MBSI)-related cis-elements (**Figure 7**).

According to our results, the promoters of *DlMGT9*.2 and *DlMGT10* contained palisade mesophyll cells-specific HD-Zip 1 cis-acting elements. **Figure 6F** shows that *DlMGT10* is highly expressed in leaves than in roots. The expression of *DlMGT10* in leaves increased significantly under magnesium deficiency stress. This result suggests that the HD-Zip 1 cis-acting element in the *DlMGT10* promoter may be closely related to its specific response to Mg deficiency stress in leaves.

### Analysis of transcription factor binding sites in *DlMGTs* promoters

**Figure 8:**
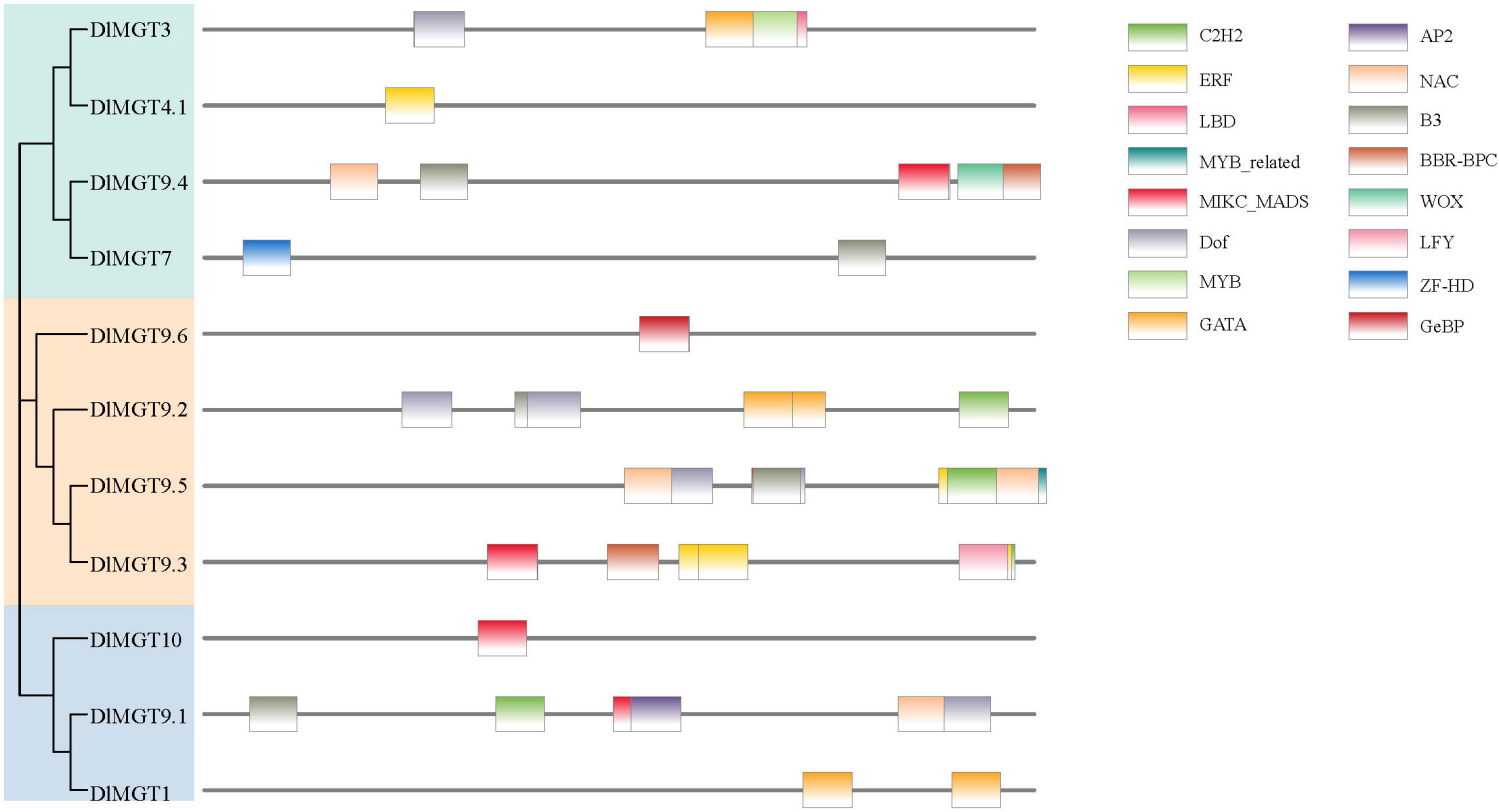
Predicted transcription factor binding sites in the promoters of *DlMGT* family genes.

We used the PlantTFDB website to predict transcription factor binding sites (TFBS) in the promoter region of *DlMGTs*. A total of 16 TFBS, including C2H2, ERF, LBD, MYB-related, MICK-MADS, Dof, MYB, GATA, AP2, NAC, B3, BBR-BPC, WOX, LFY, ZF-HD, and GeBP, were predicted in the *DlMGTs* promoter (**Figure 8**). Among the predicted results, MICK-MADS and B3 TFBS were the most numerous (12), followed by C2H2 and Dof TFBS (5) and LBD, WOX, LFY, ZF-HD, and GeBP TFBS (1). In addition, TFBS in different *DlMGTs* promoters varied significantly in type, number, and location.

### Subcellular localisation of DlMGT1

**Figure 9:**
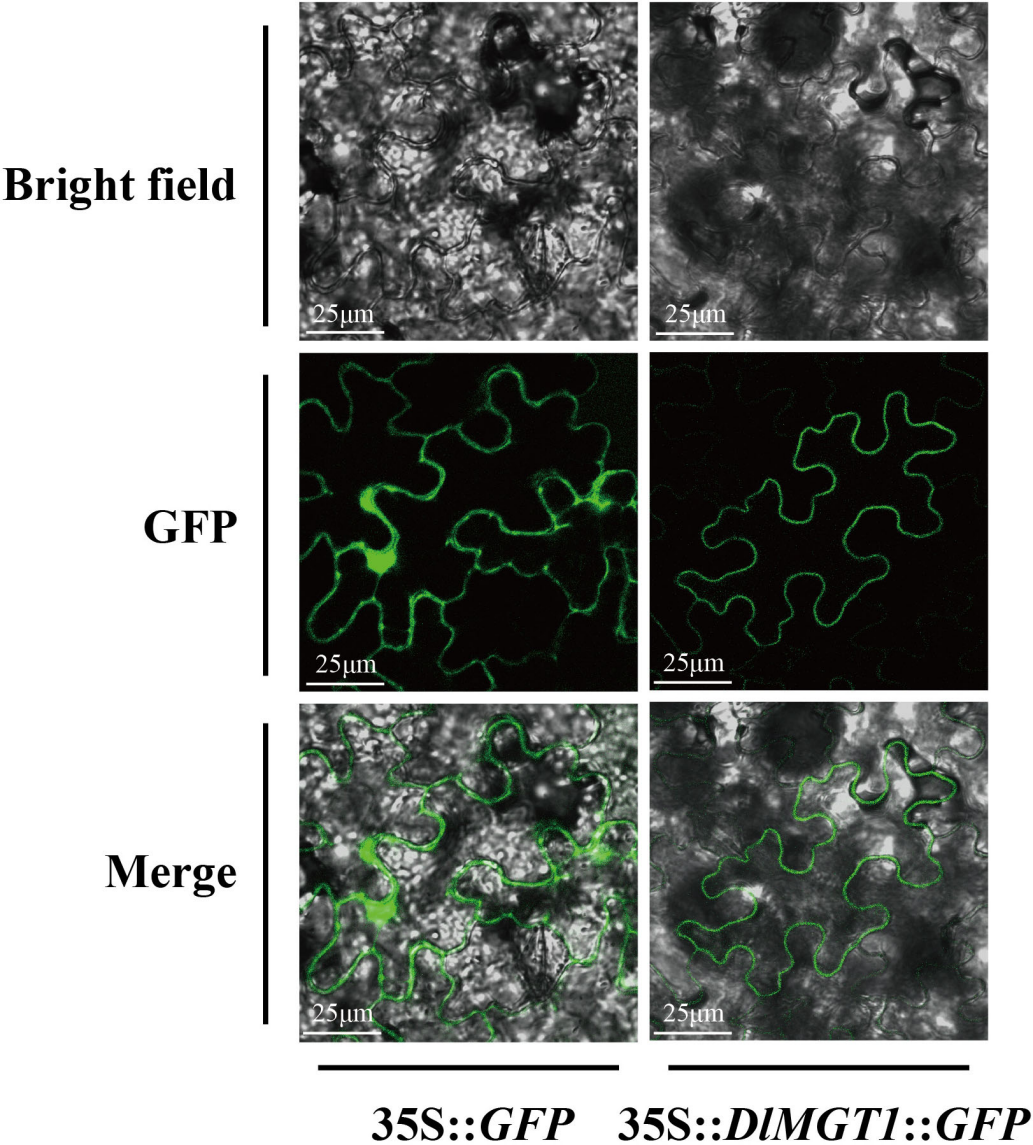
Subcellular localisation of DlMGT1 in tobacco leaves. 35S::GFP was used as a control. The scale bar length is 25 µm.

Ion transporters possess typical transmembrane domains. DlMGT1, a homologous protein of AtMGT1, was predicted to be localised in the endosomal system (**Table 1**). In order to investigate the subcellular localisation of DlMGT1, we fused *GFP* with *DlMGT1* and transiently expressed it in tobacco leaves. The results showed that DlMGT1 localised on the cell membrane in tobacco leaves, consistent with the subcellular localisation results of AtMGT1. Therefore, we speculate that DlMGT1 may have a similar physiological function with AtMGT1 in longan roots (**Figure 9**).

### Analysis of subcellular localisation of DlGATA16 and interactions with DlMGT1

**Figure 10:**
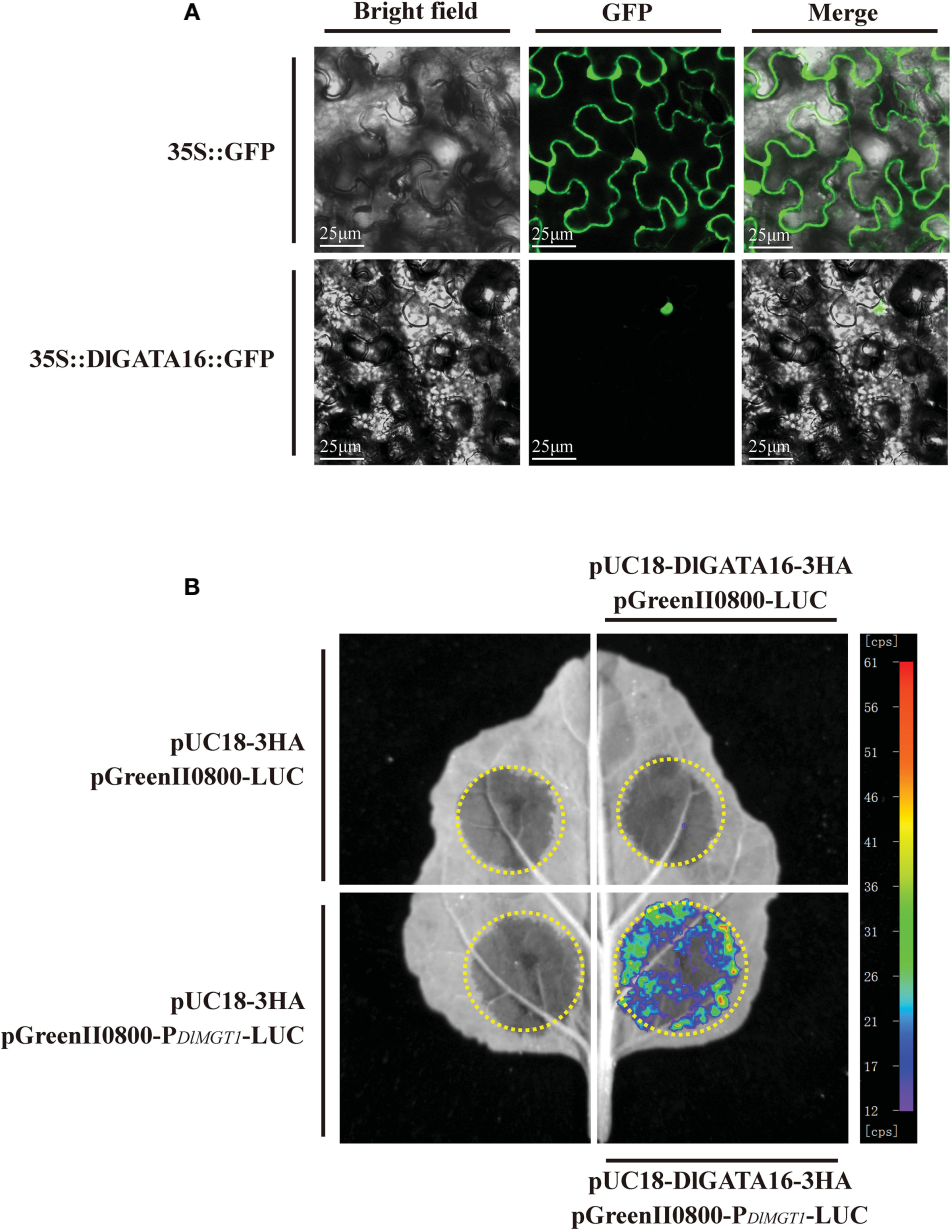
Subcellular localisation of DlGATA16 in tobacco leaves and analysis of its binding to the DlMGT1 promoter. **(A)** Subcellular localisation of DlGATA16 in tobacco leaves. 35S::GFP was used as a control. The scale bar length is 25 µm. **(B)** Dual luciferase analysis of DlGATA16 with *DlMGT1* promoter in tobacco leaves.

To further validate the regulatory role of transcription factors on *DlMGT1*, we selected GATA16, which had the highest p-value (8.8e-10) among all the predicted results of PlantTFBD (**Figure 8**). We expressed *DlGATA16* fused with *GFP*, and the pRI101-GFP empty vector was used as a control. Results showed that DlGATA16 localises in the nucleus of tobacco leaves. This result indicates that DlGATA16 is likely to function as a transcriptional regulator in the nucleus (**Figure 10A**).

The interaction between transcription factors and promoters is a prerequisite for their regulatory role. Using a luciferase assay, we performed an *in vitro* interaction analysis of the DlGATA16 with the DlMGT1 promoter. The results showed that DlGATA16 could interact with the promoter region of DlMGT1 *in vitro*. This indicates that DlGATA16 can regulate DlMGT1 expression and may be a potential transcriptional regulators of DlMGT1 (**Figure 10B**).

## Discussion

### Plant MGTs are evolutionarily conserved but still have interspecies differences

Structure variation is vital to the evolution of genes (Cao and Shi, 2012). In longan, MGTs members are grouped into two subfamilies. Subfamily members have similar intron and exon structures, and their encoded proteins consist of similar motifs (**Figure 3**). These results were also found in *Arabidopsis* (Gebert et al., 2009), rice (Saito et al., 2013), maize (Li et al., 2016), pear (Zhao et al., 2018), tomato (Regon et al., 2019), and citrus (Liu et al., 2019), suggesting that MGTs in plants are highly conserved evolutionarily. Twelve MGTs were predicted in the longan genome, more than in *Arabidopsis* (Gebert et al., 2009), rice (Saito et al., 2013), and sugarcane (Wang et al., 2019). Although the number of MGTs family members obtained in different species is different, their number may not be related to genome size. This may be due to differential amplification events during the evolution of different plant species or the evolution and variation of MGT family members that may have arisen due to the interaction between environment and genotype. There is also possible functional redundancy among *DlMGTs* genes, which needs to be investigated in more detail. The MGTs in different plants were differentiated between herbaceous and woody plants in our phylogenetic tree. Two woody plants, peach (Yu et al., 2021) and poplar (He et al., 2021), belong to different families (*Rosaceae* and *Populus*), but the PpMGTs and PtrMGTs are located on the same branch of the phylogenetic tree. Such results may imply that physiological or structural differences between herbaceous and woody plants have led to evolutionary differences in MGTs.

### The localisation of DlMGTs is more complex than other species

The intracellular localisation of transporters is crucial for their functions. Related studies indicated that AtMGTs were found to be localised in the cytoplasmic (Li et al., 2001) and vesicular (Conn et al., 2011) membranes, mitochondria (Li et al., 2008), and chloroplasts (Drummond et al., 2006). However, we found that not all DlMGTs could localise at these organelles, and four DlMGTs lacking transmembrane domains were predicted to be localised in the nucleus. Mg is an essential component for more than 300 enzymes in cells and is required for replicating and synthesizing nucleic acid (Cowan, 2022). Therefore, we suggest that DlMGTs localised in the nucleus may have different functions than others, but these biological functions remain to be confirmed experimentally. In our predicted results, six MGTs had no transmembrane domain, among which DlMGT9.4 and DlMGT9.2 were localised in chloroplasts. However, the AtMGT9 homologue DlMGT9 was predicted to be localised to the cell membrane, although this has not yet been experimentally verified. Furthermore, MGTs in chloroplasts are associated with the synthesis and metabolism of chlorophyll (Drummond et al., 2006). We suggest that DlMGT9 may have evolved to produce genetic variants that have led to changes in its localisation in the cell and may have had different functions. We next selected DlMGT1, a homolog of AtMGT1 that has been more intensively studied in *Arabidopsis*, for subcellular localisation assays. AtMGT1 was found to be localised to the root cell membrane and involved in Mg uptake and transport in *Arabidopsis* roots (Li et al., 2001). Our results show that DlMGT1 is localised on the cell membrane of *Arabidopsis* protoplasts (**Figure 8**). In terms of subcellular localization, the results of DlMGT1 and AtMGT1 were consistent, and DlMGT1 was also expressed in roots, which suggests that DlMGT1 may have partially similar biological functions to AtMGT1. The cell membrane also belongs to the inner membrane system of the cell. Therefore, the subcellular localisation result of DlMGT1 is the same as the predicted result in **Table 1**, which also verifies the accuracy of the predicted result.

### Multiple factors regulate DlMGT expression

Previous studies on CoA-type Mg transporters in bacteria and yeast have focused on the resolution of their protein crystal structures (Eshaghi et al., 2006; Lunin et al., 2006; Payandeh and Pai, 2006), whereas studies in *Arabidopsis* have focused more on the transport function of MGTs (Schock et al., 2000; Li et al., 2001; Gebert et al., 2009). Apart from a few studies on the ability of MgtB and MgtC to form operons in bacteria, no more research on the transcriptional regulation of MGTs has been conducted (Snavely et al., 1991; Tao et al., 1995). To explore the type of transcriptional regulation on MGTs, we analysed the cis-acting elements in the DlMGT promoters. Several hormones (e.g., MeJA, ABA, and SA) and stress- (e.g., drought, trauma, and low temperature) related cis-acting elements were identified in the DlMGT promoters, suggesting that the expression of DlMGTs may be responsive to multiple hormones and abiotic stresses.

### DlGATA16 may be related to chlorophyll synthesis in longan

GATA transcriptional factors play an essential role in plant growth, development, and stress response (Bi et al., 2005; Neff, 2012; Richter et al., 2013; Klermund et al., 2016). To date, little research has been conducted on GATA in plants other than *Arabidopsis*. However, the biological pathways regulated by GATA are conserved in most plants. GATA can affect not only seed germination and seedling growth (Luo et al., 2010; Behringer et al., 2014) but also plant meristematic tissue cell differentiation and leaf development (Hudson et al., 2013; Klermund et al., 2016). GATA can promote chloroplast development and chlorophyll synthesis (Chiang et al., 2012). Moreover, GATAs are highly expressed in green tissues and mediate the regulation of plastid development by cytokinins (Naito et al., 2007). Overexpression of GATA transcription factors under dark conditions promotes the differentiation of proplastids to etioplast. In the light, it promotes chloroplast development and chlorophyll production in roots (Richter et al., 2010; Kollmer et al., 2011). Likewise, MGT is essential for plant chlorophyll synthesis and leaf development (Drummond et al., 2006). It has been reported that *ZmMGT12* expression in maize is affected by light, and light-induced *ZmMGT12* expression is associated with chlorophyll biosynthesis (Li et al., 2016; Li et al., 2018). Therefore, we speculate that light-responsive DlGATA may regulate the expression of MGTs, which might play a role in chloroplast development.

## Data availability statement

The original contributions presented in the study are included in the article/**Supplementary Material**. Further inquiries can be directed to the corresponding authors.

## Author contributions

XL and HZ designed the study. JW, SH, and DH provided software and methods in data processing and analysis. JL, DG analyzed and discussed the data. All authors revised and discussed subsequent versions. All authors contributed to the article and approved the submitted version.
