# Supplementary figures and images for: Genome-wide identification of Mg2+ transporters and functional characteristics of DlMGT1 in Dimocarpus longan
Source: Front Plant Sci. 2023 Feb 2;14:1110005. doi: 10.3389/fpls.2023.1110005 (PMC9932547; doi:10.3389/fpls.2023.1110005)

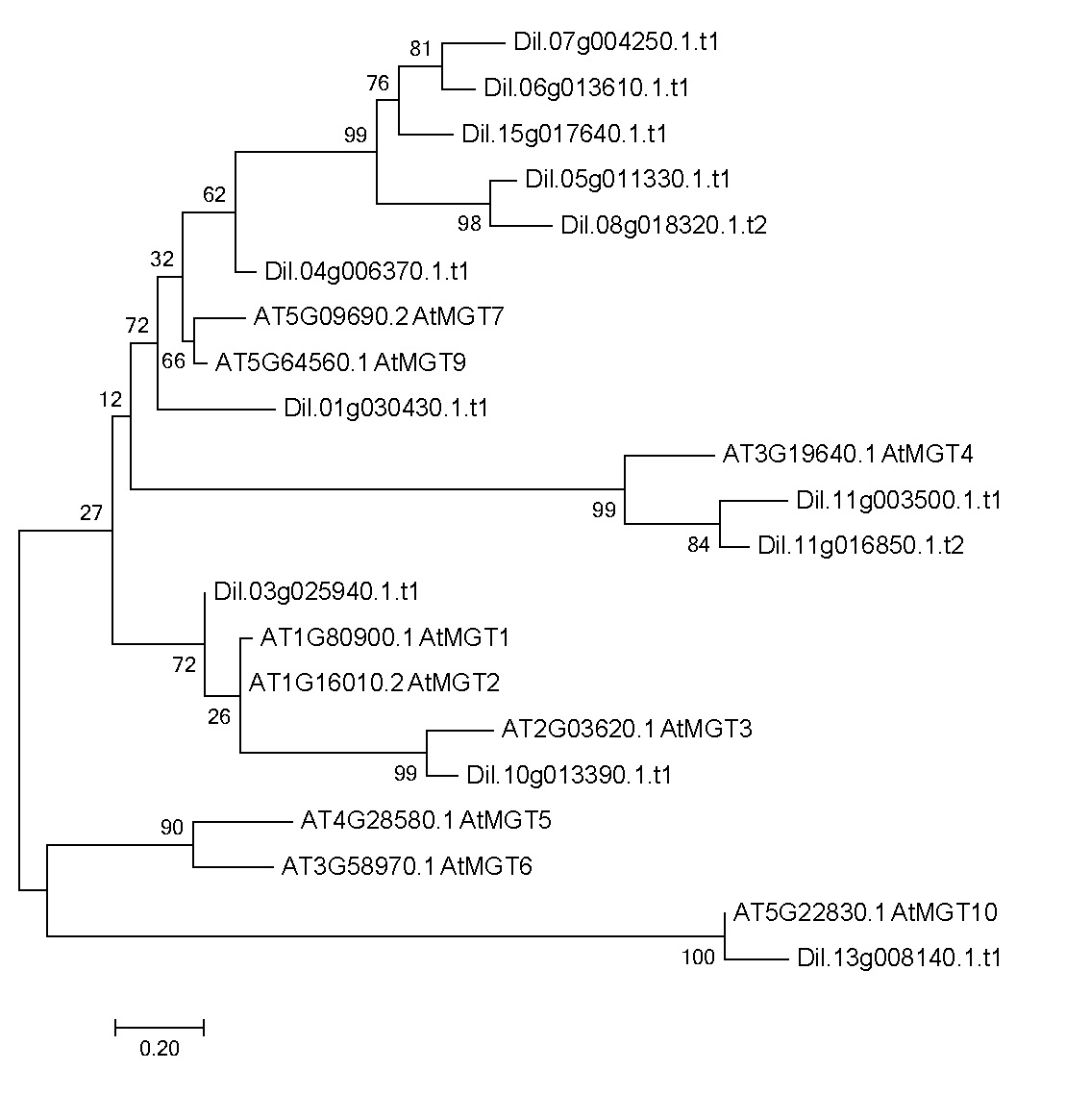

Supplement: Supplementary file 2 [file Image_1.jpeg]
